# Role of ferroptosis-related genes in prognostic prediction and tumor immune microenvironment in colorectal carcinoma

**DOI:** 10.7717/peerj.11745

**Published:** 2021-07-14

**Authors:** Chao Yang, Shuoyang Huang, Fengyu Cao, Yongbin Zheng

**Affiliations:** Department of Gastrointestinal Surgery, Renmin Hospital of Wuhan University, Wuhan, China

**Keywords:** Colorectal carcinoma, Ferroptosis, Risk signature, Prognostic prediction, Tumor immune microenvironment

## Abstract

**Background and Aim:**

Colorectal cancer (CRC) ranks the second most common cause of cancer-related mortality worldwide. Ferroptosis, a recently discovered form of programmed cell death different from other, raises promising novel opportunities for therapeutic intervention of CRC. This study intended to systematically assess the prognosis value and multiple roles of the ferroptosis-related genes in the tumor immune microenvironment of CRC.

**Materials and Methods:**

Of 1,192 CRC patients with complete information from the public datasets (TCGA CRC, GEO GSE39582 and GSE17538 cohorts) were selected for analysis. Firstly, K-means consensus clustering was performed to identify ferroptosis-associated subtypes in CRC patients. Subsequently, we constructed a risk signature based on ferroptosis-related genes in TCGA cohort and acquired its validation in two GEO cohorts. Additionally, we established a nomogram integrating the risk signature and clinical factors to improve risk assessment of CRC patients.

**Results:**

Five molecular subtypes were identified by consensus clustering for ferroptosis-related genes. There were significant differences in the overall survival, immune cells infiltration status and PD1/PD-L1 mRNA among the five clusters. Then, a risk signature based on the ten-gene was constructed which could distinguish effectively high-risk group among CRC patients in both training and validation sets. The high-risk patients were more likely to have an inhibitory immune microenvironment and lower stemness features. A prognostic nomogram integrated risk signature and clinicopathological features could be used as a more accurate prognostic prediction visualization tool than TNM stage alone.

**Conclusion:**

This ferroptosis risk signature may accurately differentiate between different risk populations and predict the prognosis of CRC. Besides, this study elucidated the crucial role of ferroptosis in tumor immune microenvironment.

## Introduction

Colorectal cancer (CRC) ranks third in terms of incidence among malignancies and is the second most common cause of cancer-related mortality worldwide (Dekker et al., 2019). As the most common gastrointestinal malignancy, CRC is associated with some well-known risk factors, including poor dietary patterns, obesity, alcoholic abuse, smoking and physical inactivity (Keum & Giovannucci, 2019). Besides environmental influences, divergent genomic factors also attribute to the complexity of CRC (Oines et al., 2017). Moreover, it is shown that the modality and mortality of CRC vary dramatically across different regions and countries, maybe due to socioeconomic factors (Carr et al., 2018). With the high-level heterogeneity, CRC presents huge difficulty in its prevention, early detection and treatment. Therefore, comprehensive and accurate risk assessment are particularly critical for diagnosis and surveillance of CRC. Currently, the approaches of risk assessment and monitoring of CRC depend largely on clinical features and pathological parameters. Tumor-node-metastasis (TNM) staging system is the most widely-used risk assessment tool (Weiser, 2018). Nevertheless, the current TNM staging system presents notable limitations in dealing with prognostic prediction on such a highly heterogeneous disease. Transcriptome profiling was extensively used in characterizing prognostic signatures of CRC patients, and obtained favorable effect in clinical application (Wang et al., 2020; Tokunaga et al., 2020). Hence, a robust prognostic genomic-clinicopathologic signature may contribute to more accurate individualized survival prediction.

Ferroptosis is a recently discovered form of programmed cell death characterized as iron-dependent accumulation of lethal lipid peroxidation (Tang et al., 2018; Mou et al., 2019). There is a fundamental difference in molecular mechanism between ferroptosis and other cell death, including apoptosis, necrosis and autophagy (Gao et al., 2016). Ferroptosis is morphologically manifest by smaller mitochondria with condensed membrane and reduced or vanished cristae (Dong et al., 2019; Park & Chung, 2019). Increasing evidences show that ferroptosis serves a crucial role in cancer cell death among various cancer types including CRC (Chen et al., 2020; Guo et al., 2018). Some small molecule inducers of ferroptosis, including, Erastin, Sulfasalazine, FIN56 and Sorafenib, showed promising outcomes of anti-tumor treatment especially in some chemoresistance conditions (Liang et al., 2019). Apart from ferroptosis inducers, numerous genes also have been identified as modulators or markers of ferroptosis (Zhou et al., 2020). Previous studies reported that enhanced expression of HMOX1 was found involved in Erastin-induced ferroptosis and up-regulation of GCLM also contributed to the promotion of ferroptosis in CRC (Sharma et al, 2020). Furthermore, ferroptosis mediated by HO-1 hyper-expression was interrogated in a human colon cancer cell line (Malfa et al., 2019). On the contrary, the well-known tumor suppressor gene P53 was related to the downregulation of metabolic stress-induced ferroptosis (Kang, Kroemer & Tang, 2019). Both apoptosis evasion and enhancement of anti-apoptotic ability are considered to be important mechanisms of chemotherapy resistance in CRC (Hu et al., 2016; Mohammad et al., 2015). As a programmed cell death method completely differs from apoptosis, ferroptosis is expected to provide new hope for tumor treatment. Previous studies have reported prognostic models based on key gene expression profiles, which provide valuable supplementary information for the TNM staging system (Tokunaga et al., 2020). Nevertheless, the role of ferroptosis-related genes (FRGs) in prognostic prediction and tumor microenvironment remains largely unknown.

This study intended to systematically assess the prognosis value and multiple roles of the FRGs in the tumor immune microenvironment. In the study, we downloaded mRNA expression profiles and clinical data of CRC patients from public databases, the Cancer Genome Atlas (TCGA) and Gene Expression Omnibus (GEO). FRGs-associated subtypes in CRC patients were identified to improve prognostic risk stratification by K-means consensus clustering. Subsequently, we constructed a risk signature based on FRGs in TCGA cohort and acquired its validation in two GEO cohorts. Additionally, we established a nomogram integrating the risk signature and clinical factors to ameliorate prognostic assessment of CRC patients. This study also provided insights into the immune landscape and stemness feature among different risk groups. Collectively, this FRGs signature and nomogram might provide effective prediction tool and insight into novel potential intervention strategies for CRC.

## Materials and Methods

### Data acquisition

**Figure 1:**
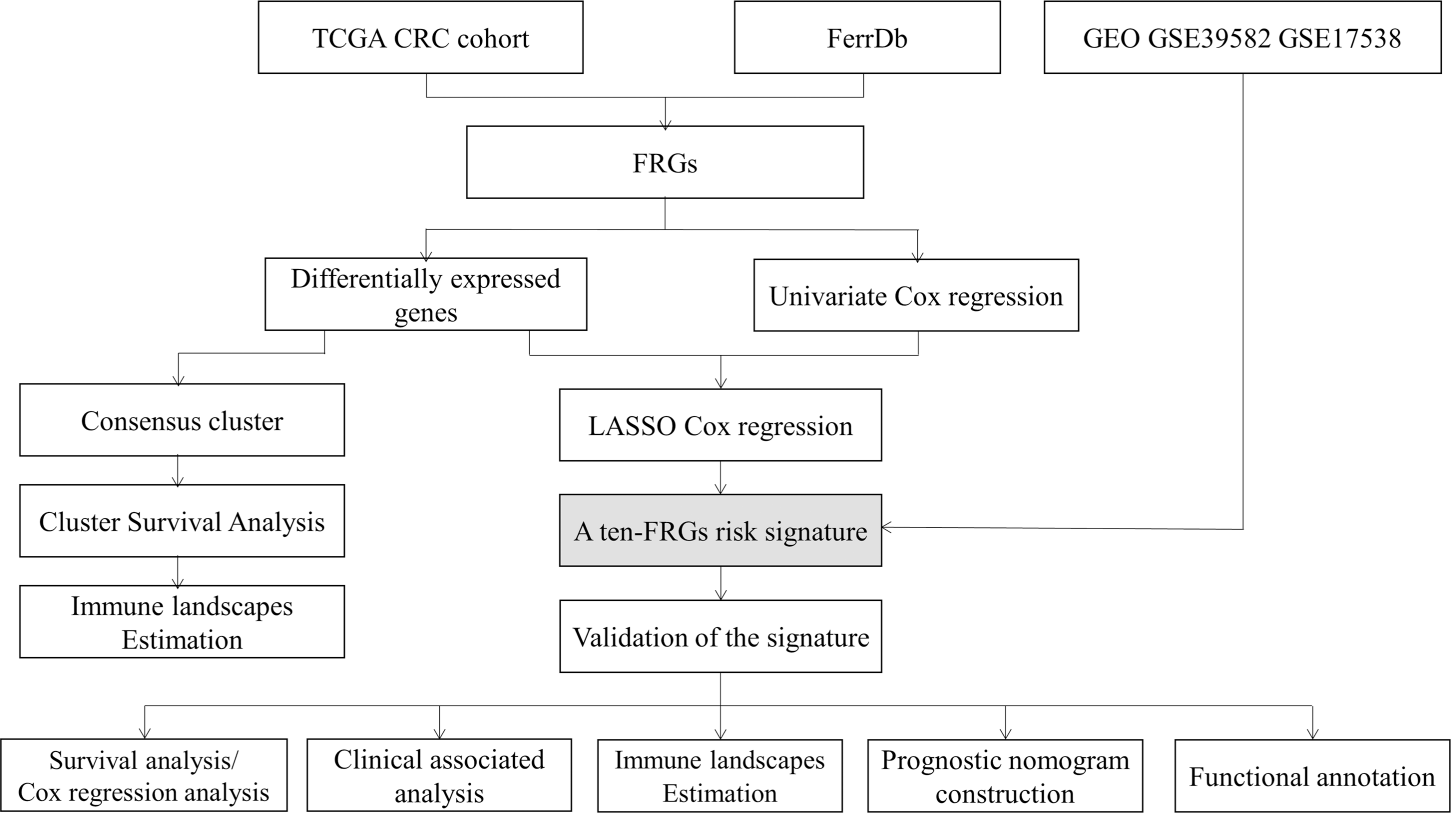
Flow diagram of the study. (FRGs: ferroptosis-related genes).

The flow chart of this study is shown in Fig. 1. In training set, the level 3 RNA sequencing (RNA-seq) and matched clinical data of patients with CRC were downloaded from TCGA CRC cohort (https://portal.gdc.cancer.gov/repository). In validation set, the raw CEL data and corresponding clinical features of CRC patients were obtained from GEO GSE39582 and GSE17538 cohorts (https://www.ncbi.nlm.nih.gov/geo/).

The exclusion criteria of this study were set as follows: (1) Patients without complete clinical information. (2) Patients whose clinical follow-up time were less than 30 days. Consequently, 481 cases (437 tumor and 44 normal samples) conforming to the criteria were included in the training group, 542 cases (523 tumor and 19 normal samples) in GSE39582 and 238 cases (232 tumor and six normal samples) in the GSE17538 cohort (Supplementary table1). The FPKM format of RNA-seq data from TCGA cohort was converted into log_2_(TPM+1) for normalized counts. The robust multi-array average (RMA) algorithm was employed to normalize the raw data from GEO cohorts by the *affy* R package (Gautier et al., 2004). Both TCGA and GEO databases are publicly available, thus ethical approval is not required for present study.

### Identification of FRGs

FerrDb is an online database containing information about regulators and markers of ferroptosis-related diseases (Zhou & Bao, 2020). A total of 214 FRGs that have been verified in homo sapiens were downloaded from FerrDb. The expression data of 200 FRGs were eventually matched in the TCGA and GEO cohorts for subsequent analysis (Table S2).

### Establishment of the risk signature

The differentially expressed genes (DEGs) between tumor and adjacent normal tissues were identified in TCGA cohort with a false discovery rate (FDR) <0.05 using *limma* R package (Ritchie et al., 2015). Then, the univariate Cox analysis was used to find out FRGs with prognostic value among DEGs. Then STRING database was employed to build an interaction network of those DEGs (http://string-db.org/). Afterwards, LASSO Cox regression analysis was performed to construct a prognostic model with minimizing the risk of overfitting. Variables selection were processed through LASSO algorithm by *glmnet* R package (Engebretsen & Bohlin, 2019). The risk score of the patients is calculated according to the normalized expression level of each gene and corresponding regression coefficient. The formula is established as follows: Risk Score = Σ *β*FRGs × ExpFRGs. (where *β* is the coefficient of FRGs and Exp is the expression level of the FRGs).

### Estimation of the risk signature for patient prognosis

The CRC patients were classified into high- and low-risk groups according to the median value of the risk score. Survival curves between two groups were drawn by the *survival* and *survminer* R packages and compared with the *Log-Rank* test. Then, the area under the curves (AUCs) of time-dependent receiver operating characteristic (ROC) curves were calculated for predicting 1-year, 3-year and 5-year overall survival (OS). To exploration whether the risk signature could be used as an independent factor of OS in CRC, univariate and multivariate Cox regression analysis were performed. The relationship between the risk score and clinicopathological features was analyzed by the Wilcoxon rank sum test.

### Construction and validation of a predictive nomogram

Nomogram was structured combining all independent prognostic factors from the previous step employing *rms*, *foreign*, and *survival* R packages (https://CRAN.R-project.org/package=rms, https://CRAN.R-project.org/package=foreign). The calibration curves and ROC curves were used to evaluate the capability of calibration and discrimination of the nomogram, respectively.

### Functional enrichment analysis

The Gene Ontology (GO) and Kyoto Encyclopedia of Genes and Genomes (KEGG) analysis were conducted based on the DEGs between different risk groups using *clusterProfiler* R package (Yu et al., 2012).

## Results

### Consensus clustering of FRGs associated with the OS and distinct immune cell infiltration

**Figure 2:**
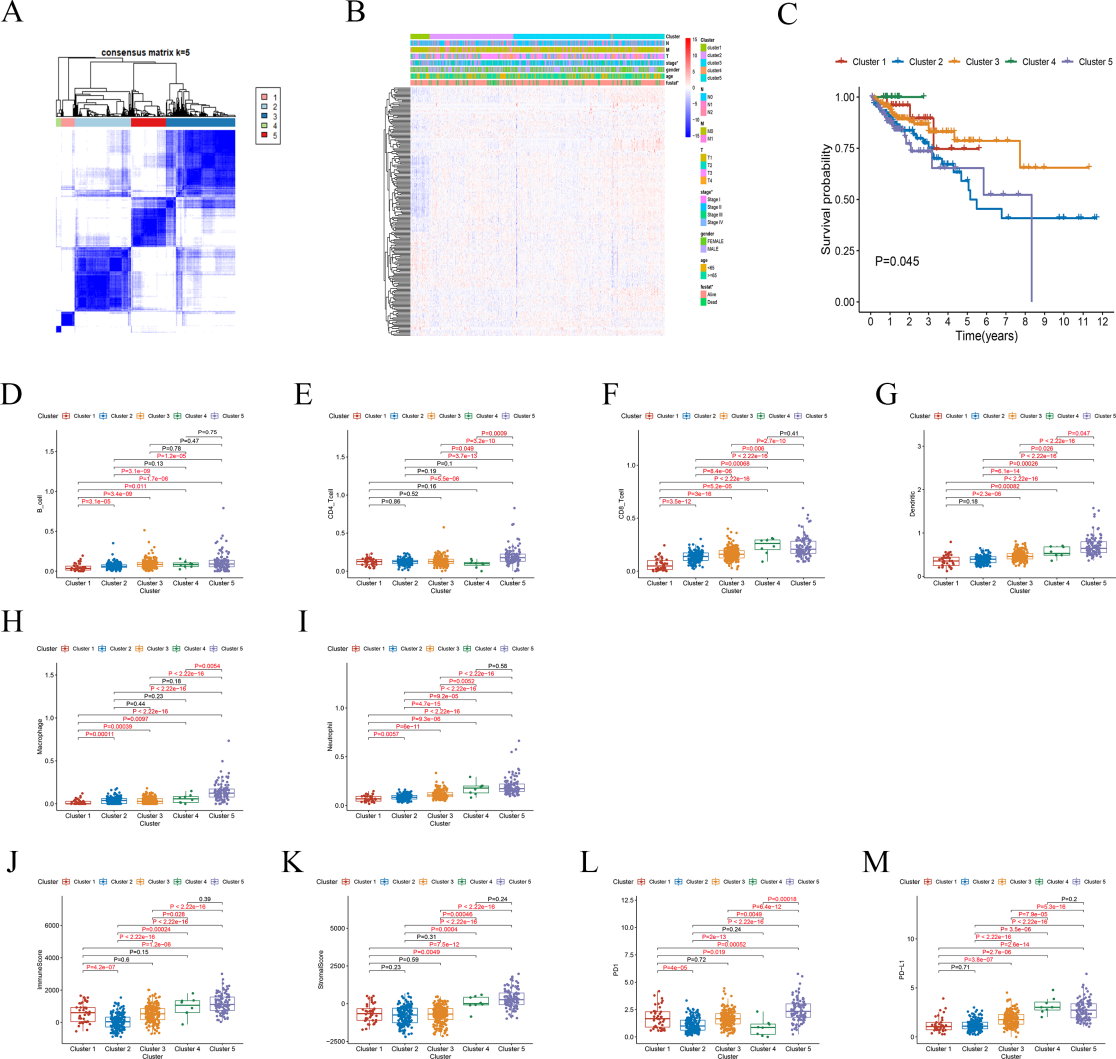
Differential clinicopathological parameters and immune landscape of CRC in clusters. (A) Consensus clustering matrix for *k* = 5. (B) Heatmap and clinicopathologic features of the five clusters. (C) Kaplan–Meier curve of overall survival among five clusters. (D–I) Different abundance of immune cell among the five clusters. (J–K) The ImmuneScore and StromalScore significantly differ between the five clustered groups. (L–M) The expression levels of PD1 and PD-L1 mRNA among the five clusters.

In following step, 437 tumor samples were clustered using the *ConsensusClusterPlus* R package after removing normal samples (Wilkerson & Hayes, 2010). Clustering was performed based on the expression levels of the 200 FRGs using the K-means clustering algorithm. Considering the stability of clustering, the *k* = 5 seemed to be an optimal clustering patterns from *k* = 2 to 9 according to the expression similarity of FRGs (Fig. 2A and 2B). Finally, the CRC patients in training group were clustered into five subtypes. There are significant differences in the OS among the five clusters (*P* = 0.0455, Fig. 2C). Subsequently, the abundance of 6 immune cells among five groups was also analyzed. The abundance of six immune cells, including B_cell, CD4_T cell, CD8_T cell, Neutrophil, Macrophage and Dendritic, was downloaded from the TIMER website (Li et al., 2017). The ImmuneScore and StromalScore were calculated by ESTIMATE algorithm to estimate the infiltration levels of immunes and stromal cells in CRC (Yoshihara et al., 2013). The results showed that there are markedly different abundance of immune cells, PD1 and PD-L1 mRNA among five clusters (Figs. 2D–2M).

### Identification of prognosis-related DEGs

**Figure 3:**
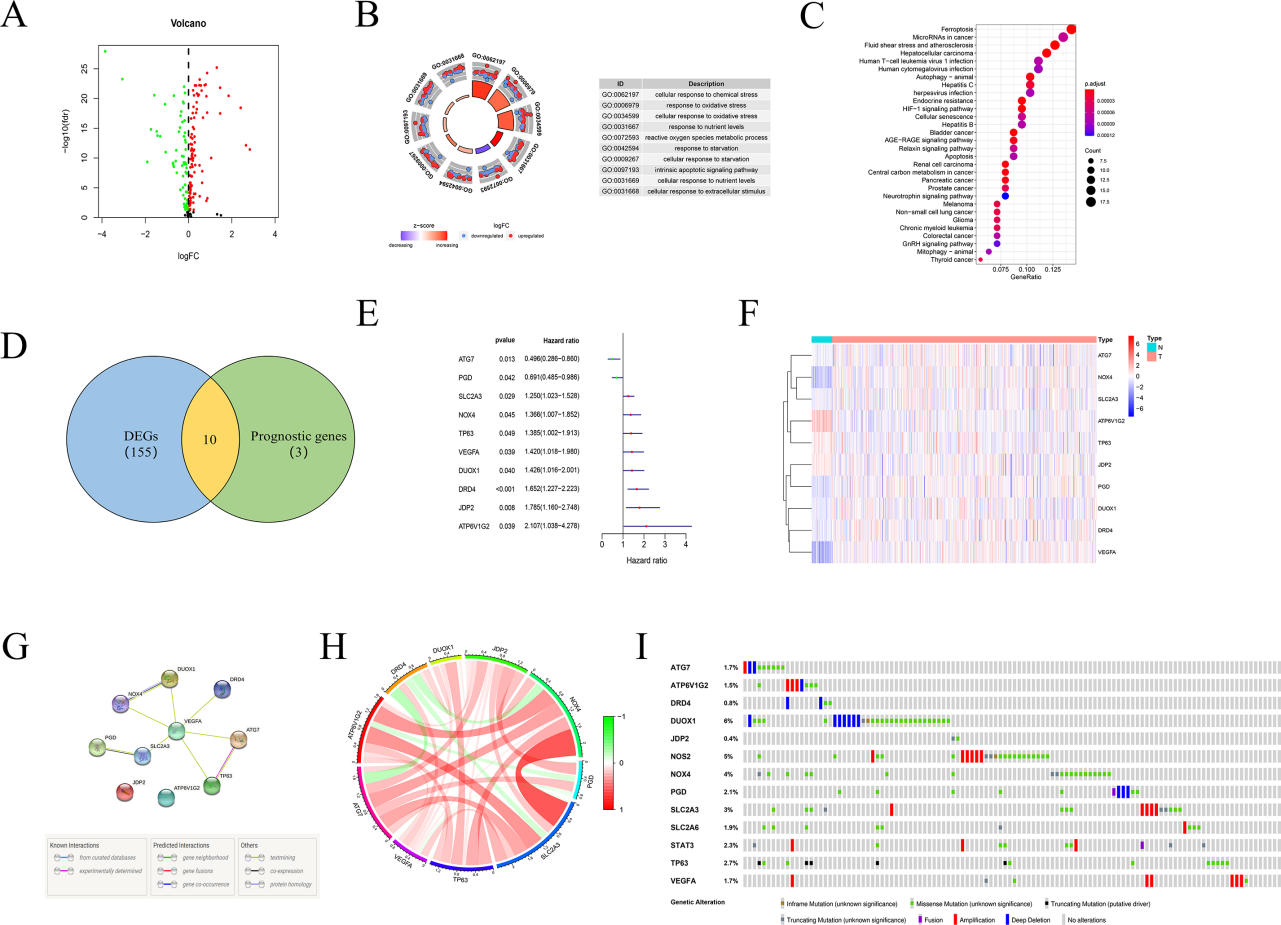
Identification of prognosis-related key FRGs in CRC. (A) Volcano map of DEGs between normal and tumour tissues. Red represented up-regulated genes and green represented down-regulated genes. (B, C) GO and KEGG pathway enrichment analysis of these 165 DEGs. (D) Venn diagram for the shared genes between prognosis-related FRGs and DEGs. (E) The result of the univariate Cox regression analysis of these shared genes. (F) Heatmap of these shared genes. (G) Protein protein interaction (PPI) network of these genes. (H) The correlation between these DEGs. (I) The genetic alteration patterns of these key genes using cBioPortal.

A total of 165 DEGs between tumor and normal tissues, including 101 up-regulated and 64 down-regulated genes, were identified (Fig. 3A). Functional enrichment analysis was applied to explore the functions of these DEGs more comprehensively. The top enriched GO terms of these 165 DEGs were enriched in several basic biological processes related to extracellular stress and energy metabolism, including cellular response to chemical stress, oxidative stress, and nutrient levels and reactive oxygen species metabolic process (Fig. 3B). KEGG analysis found that these DEGs were mainly related to ferroptosis, autophagy, cellular senescence and CRC (Fig. 3C).

Univariate Cox regression analysis showed there were 13 genes with prognostic value in 200 FRGs. Finally, there were 10 FRGs associated with both differentially expressed and prognostic value by Veen analysis (Figs. 3D–3F). A protein protein interaction (PPI) network based on these genes is presented in Fig. 3G, indicating that SLC2A3, ATG7, NOX4 and TP63 were the hub genes. The correlation of these shared genes is shown in Fig. 3H. Moreover, the genetic alteration patterns of these risk-related genes were also explored to completely understand their contribution to CRC using the cBioPortal (Gao et al., 2013) (Fig. 3I).

### Establishment a prognostic risk signature

**Figure 4:**
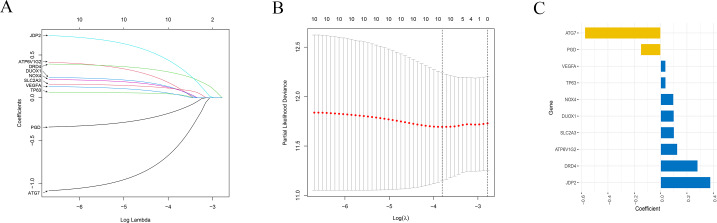
Establishment of the risk signature. (A) A LASSO coefficient profile of the 10 genes. (B) Selection of the optimal parameters in the model by 10-fold cross-validation. (C) The coefficient of each FRGs in the final signature. LASSO, Least absolute shrinkage and selection operator.

Nextly, the selected 10 FRGs were recruited into LASSO Cox regression analysis. LASSO coefficient spectrum of each gene is illustrated in Fig. 4A. The optimal parameter (lambda) was selected based on 10-time cross-validation in the LASSO model (Fig. 4B). Finally, all the 10 FRGs were included to establish the optimal prognostic risk signature. Consequently, a risk signature comprising 10 genes was developed. As shown in Fig. 4C, risk coefficient of each FRGs was calculated in the optimal model. The genes with coefficient <1, namely ATG7 and PGD, are considered as tumour suppressor genes, while the gene with coefficient >1, namely ATP6V1G2, DRD4, DUOX1, JDP2, NOX4, SLC2A3, TP63 and VEGFA, are regarded as oncogenes. The risk score of each patient is computed by the following formula both in TCGA and GEO cohorts. Risk score = (−0.578*ATG7 mRNA) + (0.128*ATP6V1G2 mRNA) + (0.282*DRD4 mRNA) + (0.101*DUOX1 mRNA) + (0.379*JDP2) + (0.098*NOX4 mRNA) + (−0.150*PGD) + (0.103*SLC2A3) + (0.039*TP63 mRNA) + (0.038*VEGFA mRNA).

### Prognostic performance of the risk signature in training and validation cohorts

**Figure 5:**
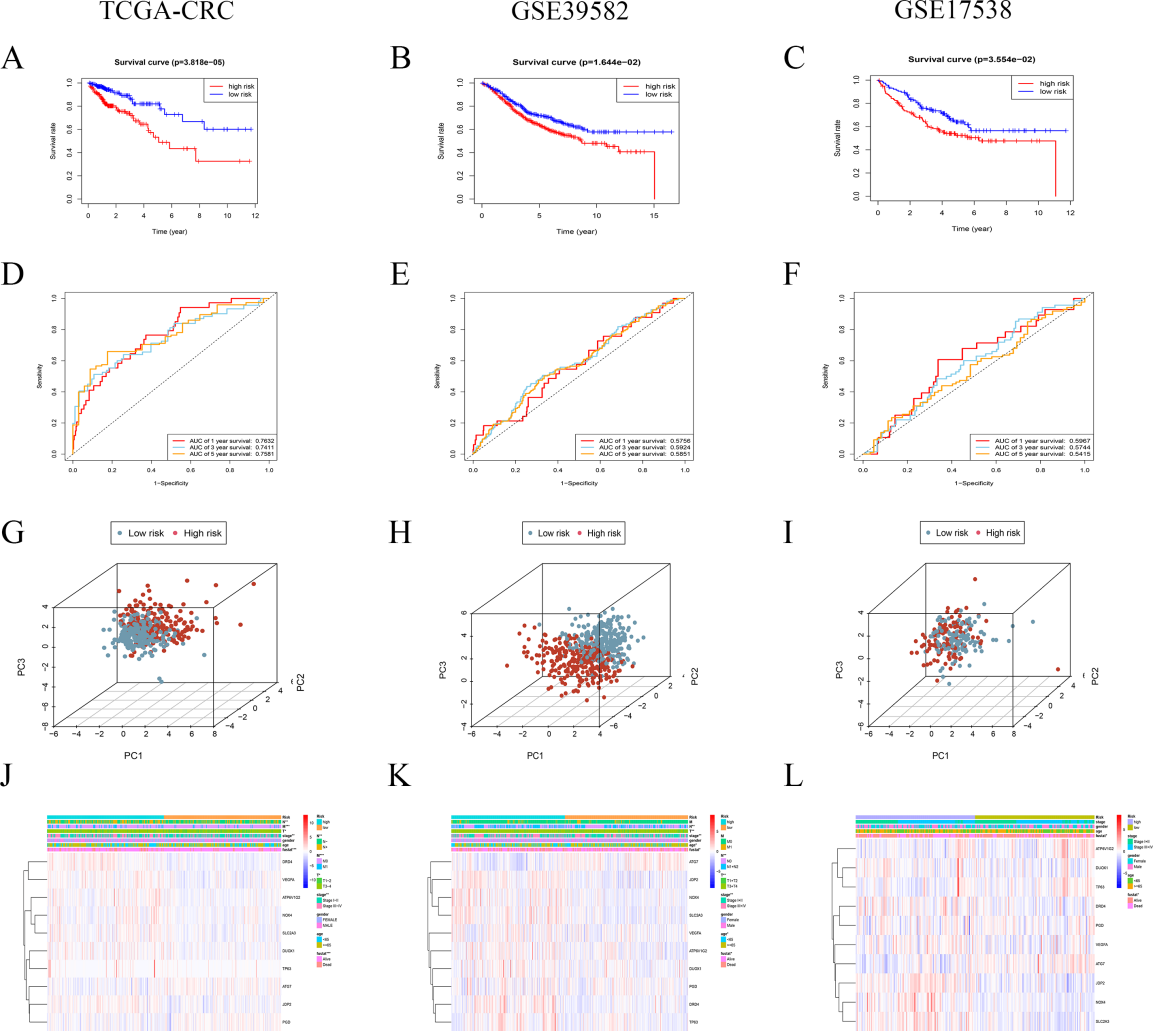
Prognostic performance of the risk signature in training and validation cohorts. (A–C) Kaplan–Meier curves of the risk signature for CRC patients with different risk groups in TCGA CRC, GSE39582 and GSE17538 cohort, respectively. (D–F) Time-dependent ROC analysis of the risk signature in TCGA CRC, GSE39582 and GSE17538 cohorts, respectively. (G–I) Principal component analysis (PCA) of the risk signature in TCGA CRC, GSE39582 and GSE17538 cohorts, respectively. (J–L) Heatmap and clinicopathologic features of different risk groups.

The patients were stratified into low- and high-risk groups based on the median value of risk score. The Kaplan–Meier survival curves indicated the patients with low risk score significantly were favorable OS than patients with high score both in TCGA and GEO cohorts (Figs. 5A–5C). The AUCs for 1-, 3-, and 5-year OS were 0.7632, 0.7411 and 0.7581 in TCGA cohort, respectively (Fig. 5D). The AUCs for 1-, 3-, and 5-year OS were 0.5756, 0.5924 and 0.5851 in the GEO GSE39582 cohort, respectively (Fig. 5E). The AUCs for 1-, 3-, and 5-year OS were 0.5967, 0.5744 and 0.5415 in the GEO GSE17538 cohort, respectively (Fig. 5F). Principal component analysis (PCA) showed that the CRC patients with high-risk could be distinguished effectively from low-risk patients using this risk signature in both training and validation sets (Figs. 5G–5I). Heatmap was used to display mRNA expression of these 10 risk-related genes. The genes with coefficient <1, like ATG7 and PGD, were more likely to have a high expression in patients with low-risk. While the patients in high-risk group were more likely to express the genes with coefficient >1, including DRD4, DUOX1, JDP2, NOX4, SLC2A3 and VEGFA (Figs. 5J, 5K and 5L).

### The risk signature is an independent prognostic factor

**Figure 6:**
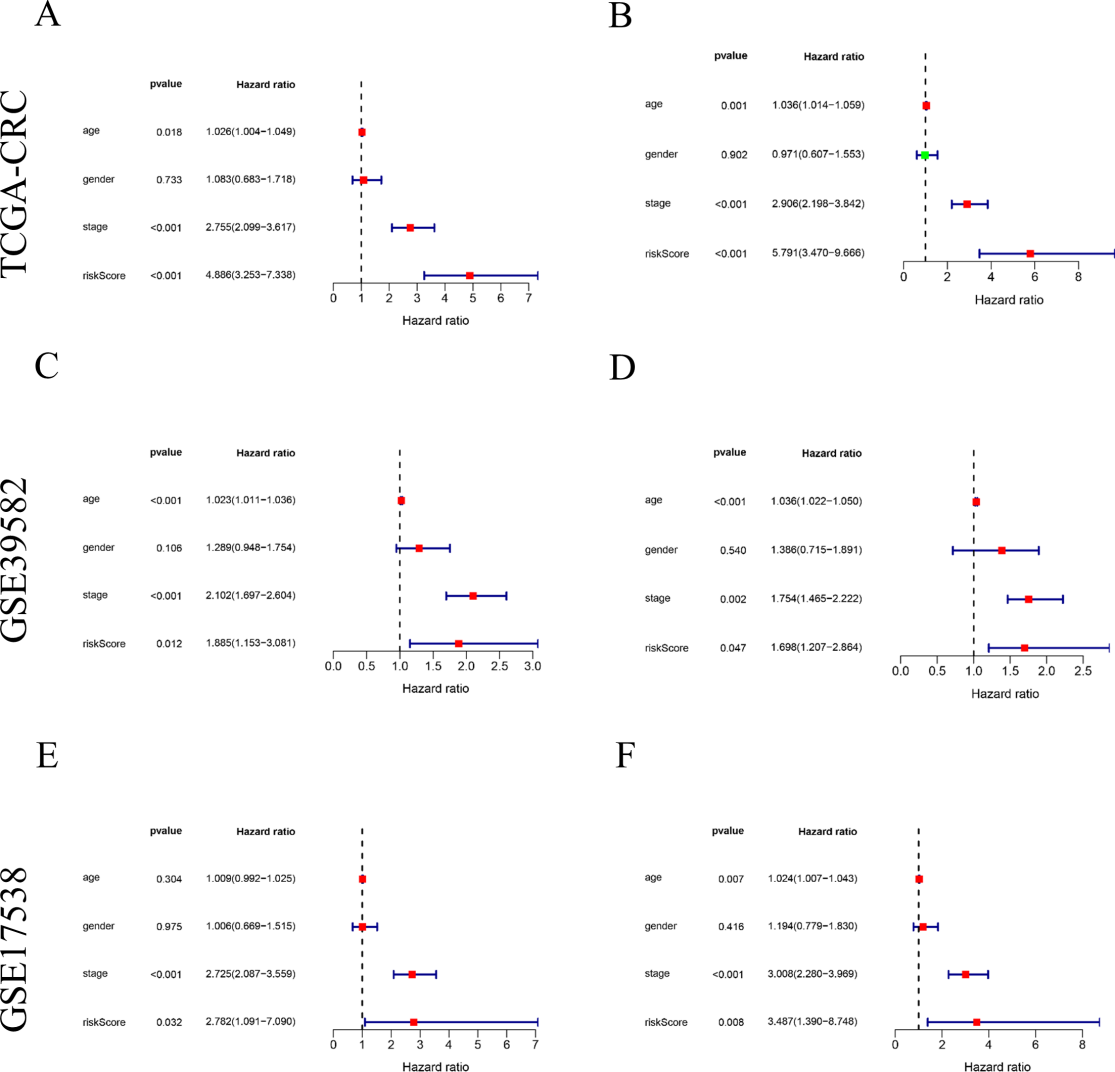
The risk signature as an independent prognostic factor both in training and validation cohorts. (A, C, E) The results of univariate Cox regression analysis in TCGA CRC, GSE39582 and GSE17538 cohorts, respectively. (B, D, F) The results of multivariate Cox regression analysis in TCGA CRC, GSE39582 and GSE17538 cohorts, respectively.

The risk score based on the ten-FRGs was indicated as an independent prognostic factor in TCGA cohort (HR = 4.886, 95% CI [3.253−7.338], *P*<0.001, Fig. 6A), in GSE39582 cohort (HR = 1.885, 95% CI [1.153−3.0814]; *P* = 0.012, Fig. 6C) and in GSE17538 cohort (HR = 2.782, 95% CI [1.091−7.090]; *P* = 0.032, Fig. 6E) by univariate Cox analysis. Meanwhile, the risk score was also significantly correlated with OS in TCGA cohort (HR = 5.791, 95% CI [3.470−9.666]; *P*<0.001, Fig. 6B), in the GSE39582 cohort (HR = 1.698, 95% CI [1.007−2.864]; *P* = 0.047, Fig. 6D) and in the GSE17538 cohort (HR = 3.487, 95% CI [1.390−8.748]; *P* = 0.008, Fig. 6F) by multivariate Cox analysis.

### Relationships between the risk score and clinicopathological features

**Figure 7:**
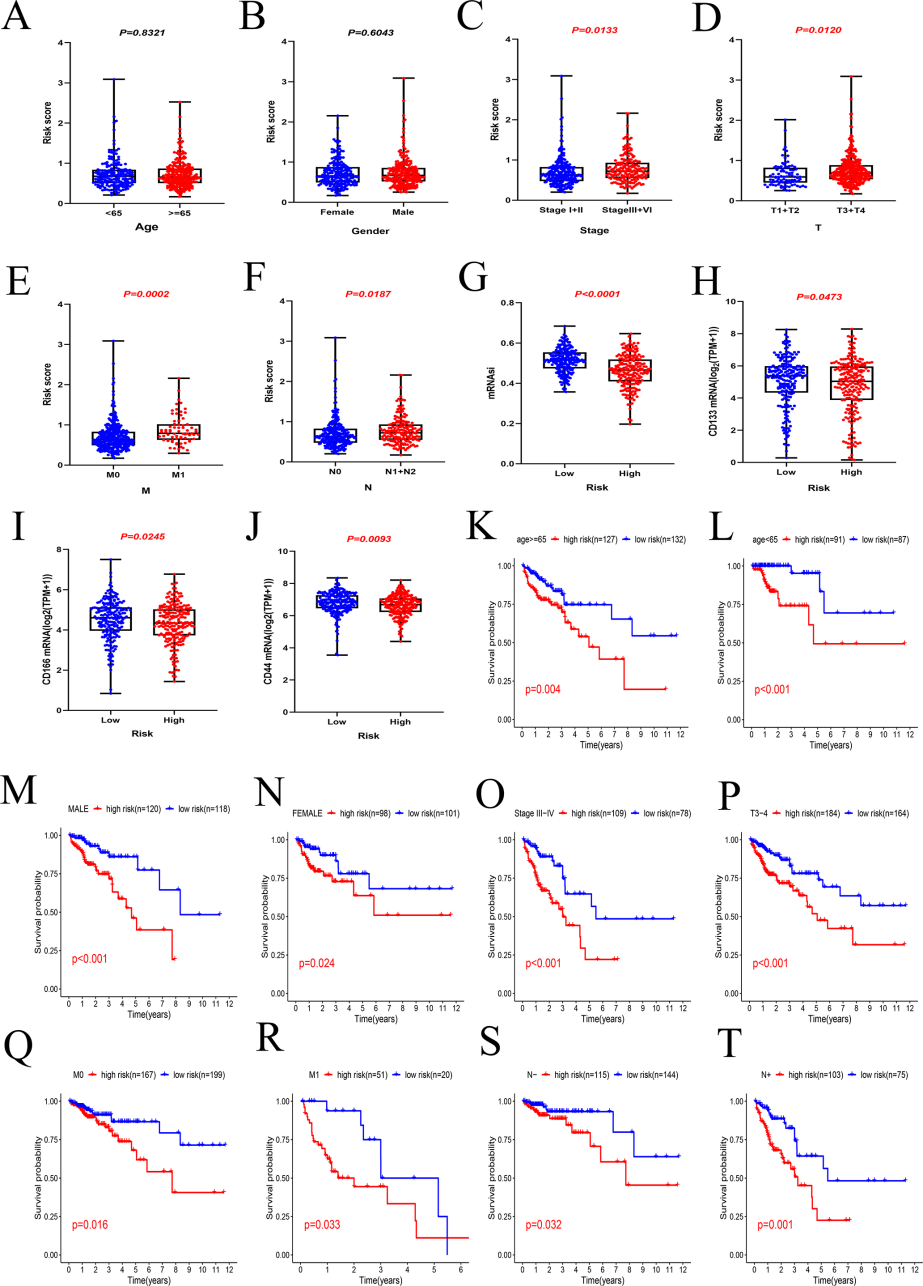
Relationships between the risk score and clinicopathological features. (A)(B) The risk score was independent with age and gender. (C–F) The risk score was correlated with pathological features in CRC, including tumour stage, pT stage, pM stage and pN stage. (G–J) The patients in low-risk group with higher stem cell characteristics, including mRNAsi, CD133, CD166 and CD44 mRNA. (K–T) The result of subgroup survival analysis.

The results showed that the risk score was independent with age (*P* = 0.8321, Fig. 7A) and gender (*P* = 0.6043, Fig. 7B). It was discovered that the risk score was correlated with pathological features in CRC. The risk score in stage III + IV patients was higher than those in stage I + II (*P* = 0.0133, Fig. 7C). The same results were obtained in other groups, including pT stage (T3 + T4 vs. T1 + T2, *P* = 0.0120, Fig. 7D), pM stage (M1 vs. M0, *P* = 0.0002, Fig. 7E) and pN stage (N1 + N2 vs. N0, *P* = 0.0187, Fig. 7F).

In addition, the relationship between risk score and cancer stem cells (CSCs) characteristics was also investigated in this study. The mRNA expression-based stemness index (mRNAsi) is widely used to evaluate stem cell characteristics of tumor cells. In this study, the mRNAsi of each CRC patient in TCGA cohort was downloaded from previous study of Malta et al. (2018). It was clear that the patients in low-risk group usually have higher stem cell characteristics, including mRNAsi (*P* < 0.0001, Fig. 7G), CD133 mRNA (*P* = 0.0473, Fig. 7H), CD166 mRNA (*P* = 0.0245, Fig. 7I) and CD44 mRNA (*P* = 0.0093, Fig. 7J).

The results of subgroup survival analysis indicated that the risk signature remains an independent prognostic factor in age (age ≥ 65 or <65, Figs. 6K and 6L), gender (male or female, Figs. 6M and 6N), tumor stage (III + IV, Fig. 6O), T stage (T3 + T4, Fig. 6P), M stage (M0 and M1, Figs. 6Q and 6R) and N stage subgroups (N0 and N1+N2, Figs. 6S and 6T). It is obvious that the low-risk patients present markedly longer OS than high-risk patient in any clinicopathological subgroups.

### Integrated a prognostic nomogram by combining risk signature and clinicopathological features

**Figure 8:**
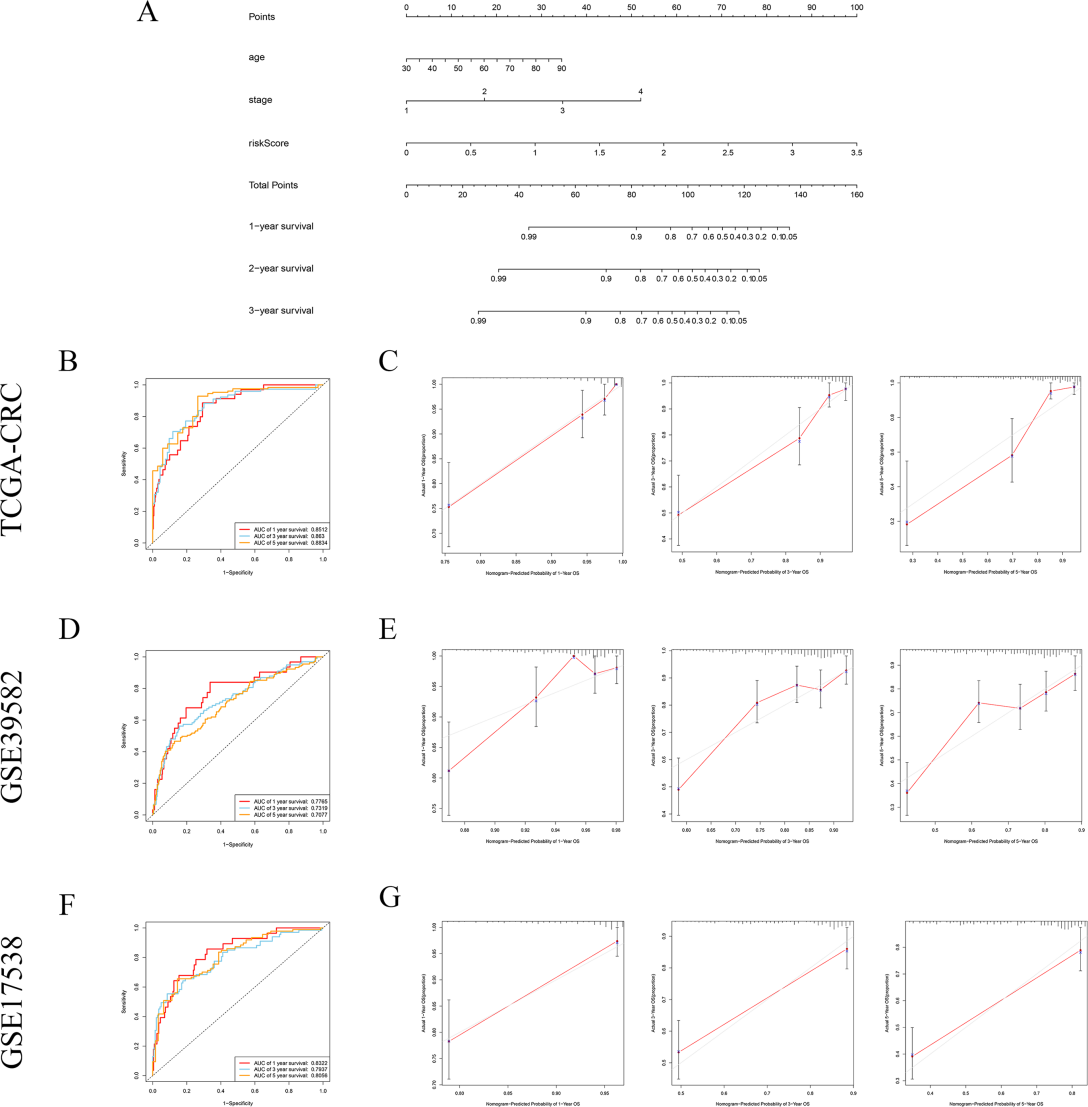
Integrated prognostic nomogram by combining risk signature and clinicopathological features. (A) A genomic-clinicopathologic nomogram predicting 1-, 3-, and 5-year OS of CRC. (B) (C) Time-dependent ROC curves and calibration plots of the combining nomogram in training set. (D) (E) Time-dependent ROC curves and calibration plots of the combining nomogram in the GSE39582 cohort. (F) (G) Time-dependent ROC curves and calibration plots of the combining nomogram in the GSE17538 cohort. OS, overall survival.

Nomogram is a powerful visualization tool in clinical practice for calculation individual risk value by integrating multiple factors. In this study, the nomogram containing four factors, namely age, gender, disease stage and risk score, was established for predicting the OS in CRC patients. A certain score is assigned to each factor based on its contribution to OS. Each patient would achieve a total point from the nomogram which is significantly negatively correlated with the OS of the patient (Fig. 8A). The ROC curves indicated that the nomogram had powerful clinical application value with the AUCs of 0.8512 at 1 year, 0.8630 at 3 years and 0.8834 at 5 years in training set (Fig. 8B). Furthermore, the calibration plots suggest that the predictive probability of the nomogram match well with actual observation probability (Fig. 8C).

In validation set, the AUCs of ROC curves were 0.7765 at 1 year, 0.7319 at 3 years and 0.7077 at 5 years in the GSE39582 cohort (Fig. 8D). The AUCs of ROC curve were 0.8322 at 1 year, 0.7937 at 3 years and 0.8056 at 5 years in the GSE17538 set (Fig. 8F). Meanwhile, the calibration plots both show that the actual observed probability agrees with predictive value of nomogram (Figs. 8E and 8G).

### High ferroptosis risk score indicates an inhibitory immune microenvironment

**Figure 9:**
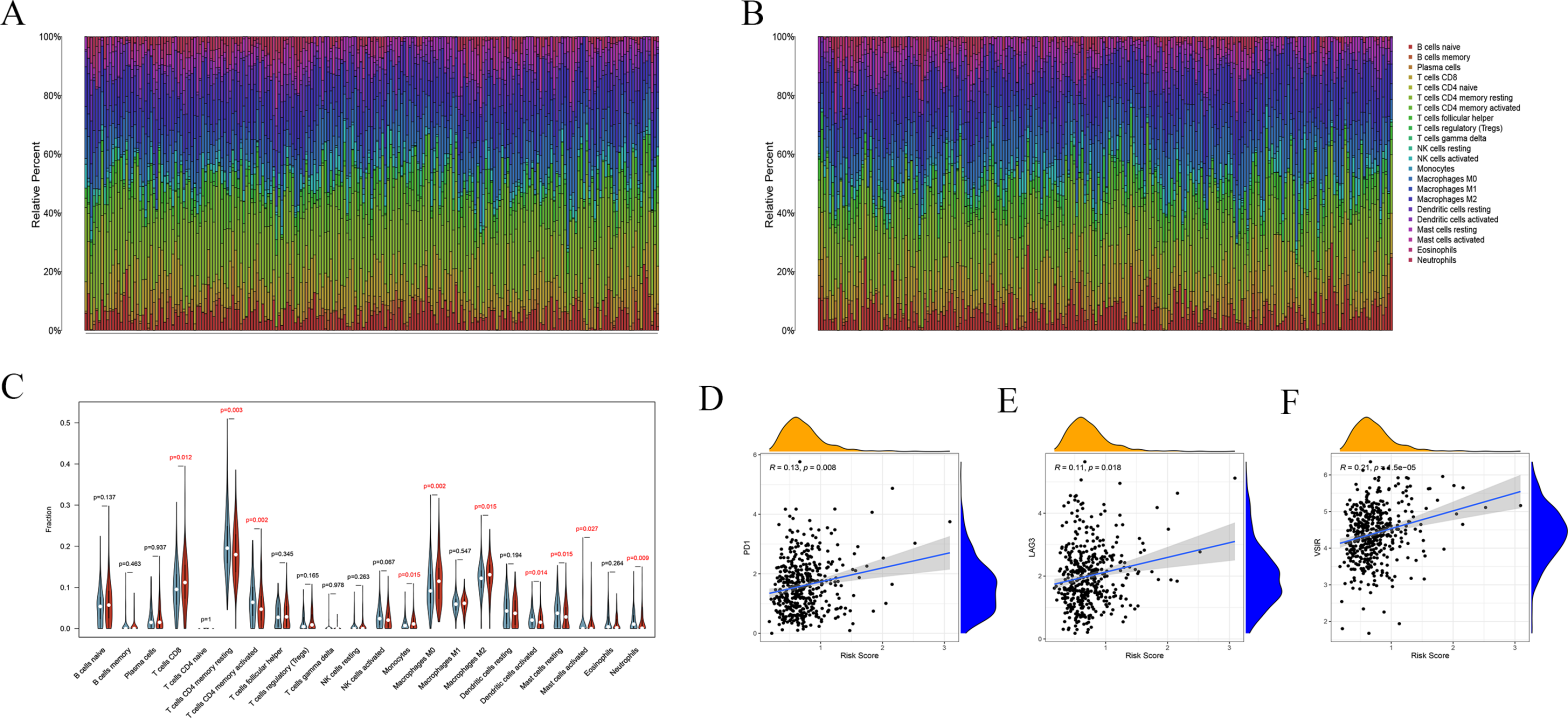
High ferroptosis risk score indicates an inhibitory immune microenvironment. (A, B) Immune landscape in high and low risk groups, respectively. (C) The distribution patterns of 22 immune cells between different groups. Green indicates the low-risk group, and red indicates the high-risk group. (D–F) Risk score was positively correlated with immune checkpoints, including PD1, LAG3 and VSIR.

The abundance of 22 immune cells was calculated by the CIBERSORT algorithm (Newman et al., 2019). There were different immune landscapes between high and low risk groups. The distribution patterns of 22 immune cells differed markedly between the high- and low-risk group (Figs. 9A and 9B). The difference analysis showed that T cells CD8 (*P* = 0.012), mast cells activated (*P* = 0.027), monocytes (*P* = 0.015), M0 (*P* = 0.002) and M2-like macrophages (*P* = 0.015) were enriched in the high-risk group distinctly, while the patients in low-risk group had higher level of T cells CD4 memory resting (*P* = 0.003), T cells CD4 memory activated (*P* = 0.002), dendritic cells activated (*P* = 0.014), mast cells resting (*P* = 0.015) and neutrophils (*P* = 0.009) (Fig. 9C).

As the markers of T cell exhaustion, immune checkpoints usually present an immunosuppressive effect in tumorigenesis and immune evasion. This study indicated the risk score was positively correlated with immune checkpoints, including PD1, LAG3 and VSIR (both *P* < 0.05, Figs. 9D–9F). These results reveal that patients with high ferroptosis risk score are more inclined to form a suppressive immune microenvironment via upregulating immune checkpoints and increasing the infiltration level of suppressive immune cells.

### Functional annotation of the risk signature

**Figure 10:**
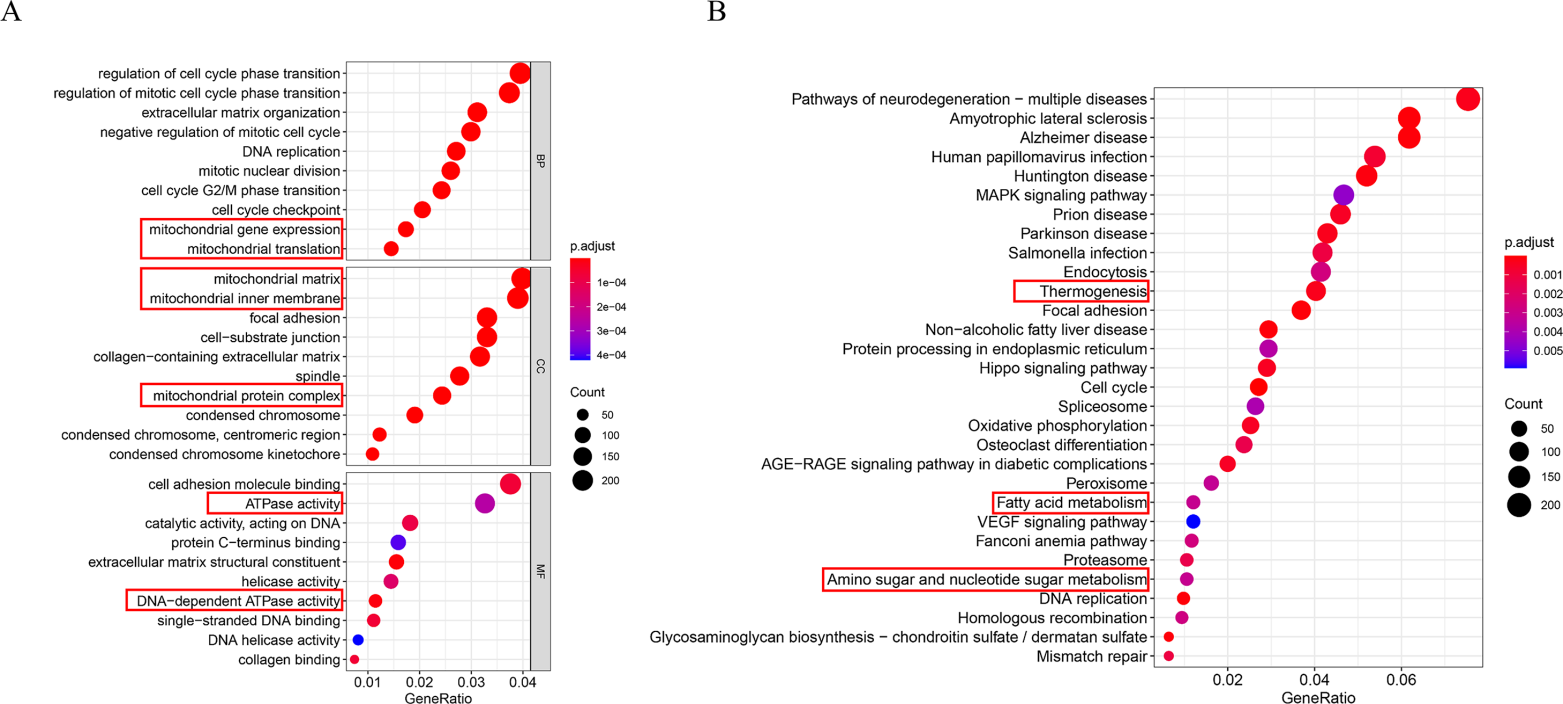
Functional annotation of the risk signature. (A) The results of GO enrichment analysis. (B) The results of KEGG pathway analysis. The red rectangles indicate the energy-related pathways, especially lipids metabolism.

The DEGs between the high- and low-risk groups were used to elucidate the molecular mechanisms by GO enrichment and KEGG pathway analysis in TCGA cohort. The results of GO enrichment analysis showed that the high-risk group is significantly enrich in energy metabolism processes, including mitochondrial gene expression, mitochondrial matrix, mitochondrial inner membrane, mitochondrial protein complex and ATPase activity (Fig. 10A). KEGG pathways enrich in the high-risk group including thermogenesis, fatty acid metabolism, and amino sugar and nucleotide sugar metabolism (Fig. 10B).

## Discussion

Ferroptosis is an iron- and reactive oxygen species (ROS)-dependency form of cell death different from other cell death and contributes to suppress tumor growth and enhancing chemosensitivity (Liang et al., 2019). Since the term of ferroptosis was raised, much effort has been devoted to its cellular mechanisms and signaling pathways. However, prognostic power of a FRGs panel for CRC has not been fully characterized. In this study, the two hundred FRGs were systematically investigated in terms of prognostic value, roles in the immune microenvironment and potential regulatory mechanisms in CRC. The majority of FRGs (165/200) were diversely expressed between normal and tumor tissues, and a total of 10 out of these genes were proved to have significantly prognostic value. This ten-FRGs risk signature was better at distinguishing patients with high risk from low risk. Furthermore, we found that the patients in high-risk group were inclined to have a suppressive immune microenvironment and lower stemness features. Meanwhile, a genomic-clinicopathologic nomogram has been developed in TCGA cohort and validated in two external cohorts. Notably, the nomogram showed superiority compared with common clinical indicators, such as TNM stage.

The ten FRGs integrated into this risk signature could be roughly classified into three categories according to the FerrDb, including driver genes (ATG7, DUOX1, NOX4 and PGD), suppressors genes (TP63) and markers genes (ATP6V1G2, DRD4, JDP2, SLC2A3 and VEGFA). ATG7 and PGD may act as protecting genes whereas ATP6V1G2, DRD4, DUOX1, JDP2, NOX4, SLC2A3, TP63 and VEGFA are the major driving force of tumorigenesis and progression. ATG7 is initially found in the process of autophagy, which participated in the formation of microtubule-associated protein 1 light chain (MAP1LC) 3 and the subsequent lipidation process, as a key step in the formation of autophagosomes (Nakamura & Yoshimori, 2017). More studies have shown that there is a close connection between autophagy and ferroptosis (Zhou et al., 2020). Iron accumulation and lipid peroxidation produced by excessive autophagy and lysosomal activity can accelerate ferroptosis (Park & Chung, 2019). Knockout of ATG7 could suppress ferroptosis through restraining Erastin-induced ferritin degradation, lipid peroxidation and iron accumulation (Hou et al., 2016). DRD4 is a member of the dopamine receptor family and involved in the positive regulation of tumor behaviors, such as tumor cell proliferation, invasion and metastasis. Erastin, an important inducer of ferroptosis, can be blocked by DRD4 leading to ferroptosis inhibition (Wang et al., 2019b).

Although the mechanism of ferroptosis has been deeply explored, the interaction between ferroptosis and tumor immune microenvironment remains unclear. It is reported that ferroptotic cancer cells can drive tumor-associated macrophage polarization through releasing oncogenic KRAS protein (Dai et al., 2020). There is a complex highly synergistic effect between ferroptosis and T cell immunity or cancer immunotherapy. Tumor necrosis factor and interferon gamma released by CD8 ^+^ T cells are the driving factors of ferroptosis (Wang et al., 2019a). Tumor clearance function of CD8 ^+^ T cells in the tumor microenvironment could be restored and enhanced by cancer immunotherapy. Mechanistically, immunotherapy-activated CD8 ^+^T cells could enhance lipid peroxidation and iron accumulation which activated ferroptosis. Meanwhile, activated ferroptosis was also capable to enhance the anti-tumor effect from immunotherapy (Wang et al., 2019a). In addition, studies have shown that CD8 ^+^ T cells can increase the sensitivity of tumor cells to radiotherapy and PD-L1/PD-1 or CTLA-4 blocking therapy by promoting ferroptosis (Lang et al., 2019). In this study, the high-risk group has higher abundance of monocytes and M2-like macrophages. Besides, the risk score is significantly positively correlated with the expression level of immune checkpoints. These mean that patients in the high-risk group tend to have a worse prognosis because they were more likely to experience immune evasion and cancer-promoting immune microenvironment. These new findings offer promising prospects for increasing antitumor effects by leveraging pro-ferroptotic activity of the immune system.

Based on the DEGs between high and low risk groups, functional analysis revealed that the biological processes and pathways about energy metabolism was markedly enriched. Tumor cells require higher levels of iron and energy metabolism (especially lipid metabolism) than normal cells to meet their rapidly proliferating needs, making them more susceptible to ferroptosis (Li & Li, 2020). There is now substantial research showing that lipid metabolism has a significant role in tumorigenesis, development, metastasis, chemoresistance and radioresistance (Rohrig & Schulze, 2016; Moloney & Cotter, 2018). Ferroptosis is a special form of programmed cell death characterized by iron-dependent ROS accumulation. ROS is a by-product of aerobic metabolism, to which lipid peroxide provides the most prominent source offering important basis for ferroptosis (Gaschler & Stockwell, 2017; Yang & Stockwell, 2016). In this study, the CSCs characteristics between different risk groups were also explored. At the cellular level, CSCs serve as an important mechanism leading to the heterogeneity and chemoresistance of CRC (Nunes et al., 2018; Li et al., 2019). Therefore, targeting CSCs has a great prospect for reversing chemoresistance. It can increase the sensitivity of colorectal CSCs to cisplatin treatment via promoting ferroptosis by knocking out or inhibiting SLC7A11 (Xu et al., 2020). In this study, the patients in low-risk group were inclined to have higher stemness feature, suggesting they are more likely to benefit from targeted CSCs therapy.

## Conclusion

We for the first time profiled the prognostic value of FRGs in CRC. Two hundred FRGs were screened and ten key prognostic genes were identified, which played a crucial function in the progression and might become the potential therapeutic targets for CRC. More importantly, a robust prognostic genomic-clinicopathologic nomogram could be used as an individualized and more accurate survival prediction tool than the TNM stage alone. Overall, this integrated study based on three CRC cohorts contribute to a deeper understanding of CRC biological behavior and potential interventions in the perspective of ferroptosis.

##  Supplemental Information

10.7717/peerj.11745/supp-1 Supplemental Information 1 Baseline characteristics of patients with CRC in training and validation cohorts

10.7717/peerj.11745/supp-2 Supplemental Information 2 200 ferroptosis-related genes in this study

## Additional Information and Declarations

### Data Availability

The following information was supplied regarding data availability: The raw transcriptome data of this study are available in the TCGA database (TCGA-COAD/READ cohorts)(https://portal.gdc.cancer.gov/) and GEO: GSE39582 and GSE17538.
