# Supplementary material for: Role of ferroptosis-related genes in prognostic prediction and tumor immune microenvironment in colorectal carcinoma
Source: PeerJ. 2021 Jul 14;9:e11745. doi: 10.7717/peerj.11745 (PMC8286063; doi:10.7717/peerj.11745)
Supplement: Supplemental Information 1 [file peerj-09-11745-s001.docx]

**Supplementary table 1:** Baseline characteristics of patients with CRC in training and validation cohorts

| Characteristic | Training Cohort | Validation Cohorts | | χ^2^ | P-value |
| --- | --- | --- | --- | --- | --- |
|  | TCGA CRC  (n=437) | GSE39582  (n=523) | GSE17538  (n=232) |  |  |
| Age |  |  |  | 4.966 | 0.0835 |
| <65 | 178 | 203 | 110 |  |  |
| ≥65 | 259 | 320 | 122 |  |  |
| Gender |  |  |  | 0.2432 | 0.8855 |
| Female | 199 | 239 | 110 |  |  |
| Male | 238 | 284 | 122 |  |  |
| Survival status |  |  |  | 47.65 | <0.001 |
| Alive | 363 | 355 | 139 |  |  |
| Dead | 74 | 168 | 93 |  |  |
| Stage |  |  |  | 11.00 | 0.0041 |
| Stage I-II | 250 | 277 | 103 |  |  |
| Stage III-IV | 187 | 246 | 132 |  |  |
| Stage_T |  |  |  | 18.93 | <0.001 |
| T1-2 | 89 | 54 | - |  |  |
| T3-4 | 348 | 469 | - |  |  |
| Stage_N |  |  |  | 1.562 | 0.2113 |
| N0 | 259 | 289 | - |  |  |
| N1-2 | 178 | 234 | - |  |  |
| Stage_M |  |  |  | 5.015 | 0.0251 |
| M0 | 366 | 464 | - |  |  |
| M1 | 71 | 59 | - |  |  |
