# Supplementary material for: Role of ferroptosis-related genes in prognostic prediction and tumor immune microenvironment in colorectal carcinoma
Source: PeerJ. 2021 Jul 14;9:e11745. doi: 10.7717/peerj.11745 (PMC8286063; doi:10.7717/peerj.11745)
Supplement: Supplemental Information 2 [file peerj-09-11745-s002.docx]

**Supplementary table 2:** 200 ferroptosis-related genes in this study

| **Genes name** |
| --- |
| CBS |
| ARNTL |
| OTUB1 |
| DRD5 |
| SLC2A6 |
| DDIT3 |
| IFNG |
| ATF4 |
| ALOX12 |
| ZFP69B |
| UBC |
| LPCAT3 |
| MIOX |
| VEGFA |
| ATG7 |
| PGD |
| NOS2 |
| STMN1 |
| DUOX1 |
| TF |
| SESN2 |
| CAPG |
| HBA1 |
| SLC3A2 |
| RB1 |
| NFS1 |
| SLC2A8 |
| TNFAIP3 |
| NOX5 |
| RIPK1 |
| PSAT1 |
| DNAJB6 |
| SLC1A5 |
| SQSTM1 |
| FADS2 |
| PLIN4 |
| CHMP6 |
| ALOX15B |
| PRKAA2 |
| OXSR1 |
| TSC22D3 |
| ALOX12B |
| MT3 |
| NOX4 |
| HSPB1 |
| ZNF419 |
| BRD4 |
| PML |
| NOX1 |
| AURKA |
| ATP6V1G2 |
| TGFBR1 |
| AGPAT3 |
| GABPB1 |
| VLDLR |
| RGS4 |
| GPT2 |
| GPX2 |
| HNF4A |
| HERPUD1 |
| MAPK14 |
| HMGB1 |
| CHMP5 |
| YY1AP1 |
| YWHAE |
| PCK2 |
| KIAA1429 |
| MT1G |
| LINC00472 |
| ELAVL1 |
| CYBB |
| NCOA4 |
| TUBE1 |
| NRAS |
| SLC7A11 |
| DPP4 |
| CA9 |
| BECN1 |
| TRIB3 |
| PRDX1 |
| HIF1A |
| GDF15 |
| SLC7A5 |
| KRAS |
| LURAP1L |
| GOT1 |
| ASNS |
| TXNIP |
| ALOXE3 |
| ATF3 |
| JDP2 |
| CD44 |
| LINC00336 |
| SLC40A1 |
| TAZ |
| ATP5MC3 |
| STAT3 |
| DDIT4 |
| G6PD |
| BLOC1S5-TXNDC5 |
| NFE2L2 |
| MYB |
| NF2 |
| CHAC1 |
| TP53 |
| IREB2 |
| CDKN1A |
| ACSL3 |
| ANGPTL7 |
| MTDH |
| ALB |
| SLC1A4 |
| SETD1B |
| IDH1 |
| AKR1C2 |
| ACVR1B |
| PHKG2 |
| NGB |
| HSPA5 |
| ALOX15 |
| SLC2A14 |
| PTGS2 |
| TXNRD1 |
| PRDX6 |
| PANX1 |
| DRD4 |
| SLC2A1 |
| FBXW7 |
| GPX4 |
| ARRDC3 |
| ZFP36 |
| CDKN2A |
| ANO6 |
| PRKAA1 |
| TFRC |
| VDAC2 |
| CEBPG |
| ZEB1 |
| RRM2 |
| SAT1 |
| SLC2A12 |
| TP63 |
| SELENOS |
| MAPK3 |
| MAFG |
| ACSF2 |
| SRXN1 |
| CISD2 |
| PROM2 |
| KEAP1 |
| AKR1C1 |
| RPL8 |
| SCD |
| LAMP2 |
| ALOX5 |
| GCH1 |
| EGFR |
| FTL |
| SLC2A3 |
| CS |
| CAV1 |
| DUOX2 |
| NNMT |
| BNIP3 |
| TMBIM4 |
| MAPK1 |
| EIF2AK4 |
| NCF2 |
| ATM |
| NQO1 |
| ISCU |
| EIF2S1 |
| AKR1C3 |
| FTH1 |
| MAP3K5 |
| LONP1 |
| BAP1 |
| ACSL4 |
| CISD1 |
| KLHL24 |
| MUC1 |
| FH |
| JUN |
| PEBP1 |
| SRC |
| HSD17B11 |
| DUSP1 |
| HIC1 |
| ABCC1 |
| HMOX1 |
| SOCS1 |
| HSF1 |
| AIFM2 |
| HELLS |
| ATG5 |
| CDO1 |
| HRAS |
| EMC2 |
| EGLN2 |
| PLIN2 |
